# A case report of nilotinib-induced irreversible interstitial lung disease

**DOI:** 10.1097/MD.0000000000028701

**Published:** 2022-01-28

**Authors:** Jun Yeun Cho, Ok-Jun Lee, Jihyun Kwon, Dohun Kim, Yoon Mi Shin

**Affiliations:** a Department of Internal Medicine, Chungbuk National University Hospital, Chungbuk National University College of Medicine, Cheongju, Korea; b Department of Pathology, Chungbuk National University Hospital, Chungbuk National University College of Medicine, Cheongju, Korea; c Department of Thoracic and Cardiovascular Surgery, Chungbuk National University Hospital, Chungbuk National University College of Medicine, Cheongju, Korea.

**Keywords:** case report, chemotherapy, drug-induced, interstitial lung disease

## Abstract

**Rationale:**

Nilotinib is a second line tyrosine kinase inhibitor to treat patients with chronic myeloid leukemia after imatinib resistance or intolerance. Drug related pulmonary complication is known to be rare. We discuss a case of nilotinib-induced interstitial lung disease presenting with nonspecific interstitial pneumonia on the unilateral lung.

**Patient concerns:**

A 46-year-old man with chronic-phase chronic myeloid leukemia presented with cough and weight loss for 2 months. He had been treated with nilotinib for 52 months.

**Diagnosis:**

Computed tomography scan showed right lung dominant consolidations, ground glass opacities and traction bronchiectasis. Bronchoalveolar lavage fluid analysis revealed no evidence of infection or malignancy. Surgical lung biopsy specimen was consistent with fibrosing nonspecific interstitial pneumonia. The patient was diagnosed with nilotinib induced interstitial lung disease.

**Interventions:**

Corticosteroid treatment was initiated with prednisolone (50 mg daily) and slowly tapered down for 2 months.

**Outcomes:**

Cough improved after the course of corticosteroid treatment. However, fibrotic lung lesions persisted. Reinitiation of nilotinib resulted in the worsening of lung lesions.

**Lessons:**

We report a case of irreversible interstitial lung disease that caused by nilotinib. Clinicians should have suspicion of this potential pulmonary complication in patients with respiratory symptoms and abnormal radiologic findings during nilotinib treatment, albeit rarely.

## 1 Introduction

Chronic myeloid leukemia (CML) is a myeloproliferative neoplasm characterized by BCR-ABL fusion gene.^[1]^ A crude annual incidence of CML is 0.7–1.0/100,000 with a median age at diagnosis of 57 to 60 years.^[2]^ Imatinib, a selective BCR-ABL tyrosine kinase inhibitor, has greatly contributed to the improvement of treatment outcome. Hochhaus et al reported that the estimated 10-year overall survival rate was 83.3% among the imatinib group without serious adverse events.^[3]^ Imatinib is generally well tolerated in patients with CML. Superficial edema, musculoskeletal pain, diarrhea and nausea are the most common adverse events with imatinib treatment.^[4]^ However, imatinib-induced interstitial lung disease (ILD) often requires discontinuation of imatinib and switch to another drug for treatment.^[5,6]^

Nilotinib is a second generation BCR-ABL Tyrosine kinase inhibitor to treat CML.^[7]^ Most commonly reported drug-related adverse events are rash, pruritus and gastrointestinal upset.^[8]^ Pulmonary hypertension and life-threatening cardiac arrhythmias can occur.^[9,10]^ Pulmonary toxicities (e.g., pleural effusion <1%) are rare, as reported in previous clinical trials.^[11,12]^ Previous case reports showed that nilotinib can successfully substitute imatinib in patients with imatinib-induced ILD.^[13,14]^ Here, we describe a rare case of nilotinib-induced ILD presenting with nonspecific interstitial pneumonia (NSIP) in the unilateral lung.

## 2 Case presentation

A 46-year-old man with chronic-phase, Philadelphia chromosome positive CML presented with chronic cough and weight loss for 2 months in our institution on September 2019. He never smoked and had no other co-morbidities or past surgical history. He was previously treated with imatinib (400 mg once daily) for 29 months from November 2009. Because of poor disease control, dasatinib (100 mg once daily) was started in April 2012. After 12 months of dasatinib, complete hematologic and cytogenetic response was achieved. Dasatinib treatment was continued to April 2015. However, dasatinib was discontinued in May 2015 due to, grade 3 or more, recurrent pleural effusion. Hence, nilotinib (300 mg twice daily) was started; he had been treated with nilotinib for 52 months.

On physical examination, he had no fever but was weak-looking. Localized crackles were auscultated on the right upper lung field. The white blood cell count was 9960 /μl (normal range 4000–10,000) with 68.9% neutrophils. C-reactive protein and erythrocyte sedimentation rates were both elevated at 8.79 mg/dl (normal range 0–0.3) and 62 mm/h (normal rage ≤9), respectively. Serologic tests for antinuclear antibody, anti-neutrophil cytoplasmic antibodies and rheumatoid factors were all negative. Lung function tests demonstrated decreased diffusing capacity of the lungs for carbon monoxide (DLCO) at 12.2 ml/mm Hg/min (55% predicted), forced expiratory volume in 1 second (FEV_1_) of 1.98 L (53% predicted) and forced vital capacity (FVC) of 2.07 L (43% predicted). A ratio of FEV_1_/FVC was 0.96.

**Figure 1:**
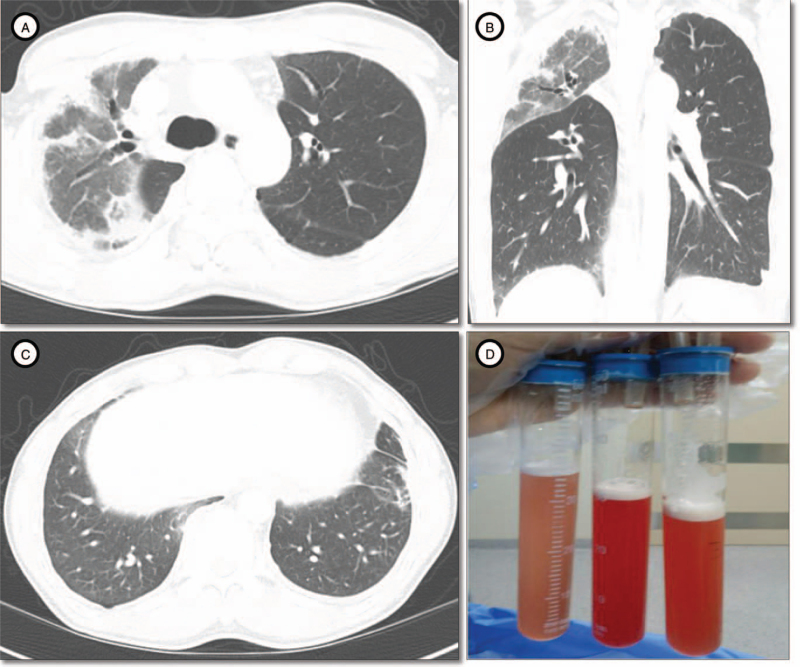
Initial computed tomographic findings and gross appearance of bronchoalveolar lavage fluid. (A, B) Computed tomography scan demonstrated consolidations, ground glass opacities, and traction bronchiectasis in the right upper lobe and the superior segment of right lower lobe. Reduced volume of the right upper lobe was noted. (C) Focal subpleural consolidation and reticular opacities were observed in the left lower lobe. (D) Bronchoalveolar lavage was performed in the anterior segmental bronchus of right upper lobe. Retrieved fluid was grossly bloody without dilution on subsequent sampling.

**Figure 2:**
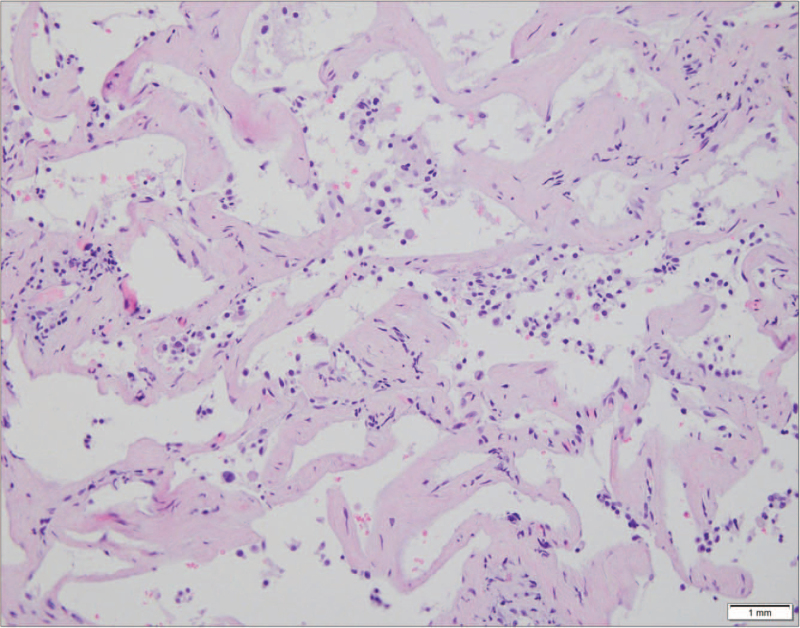
Surgical lung biopsy specimen obtained from the right upper lobe demonstrated diffuse interstitial thickening with fibrosis (hematoxylin-eosin stain, 100 × magnification).

Computed tomography (CT) scan showed consolidations, ground glass opacities, and traction bronchiectasis in the right upper lobe and the superior segment of right lower lobe. The volume of the right upper lobe was considerably reduced (Fig. 1). No mediastinal lymph node enlargement was observed. Bronchoalveolar lavage (BAL) via a flexible fiberoptic bronchoscope was performed in the anterior segmental bronchus of right upper lobe. BAL fluid was grossly bloody without dilution on subsequent sampling (Fig. 1D). Differential cell count of the BAL revealed 11% neutrophil, 38% lymphocytes and 4% eosinophil. There was no evidence of pathogenic bacteria, viruses and fungi. A polymerase chain reaction test for *Pneumocystis jirovecii* was negative. Surgical lung biopsy was performed via a video-assisted thoracoscopic surgery. Histopathology showed diffuse interstitial thickening with fibrosis, consistent with a fibrosing NSIP (Fig. 2).

**Figure 3:**
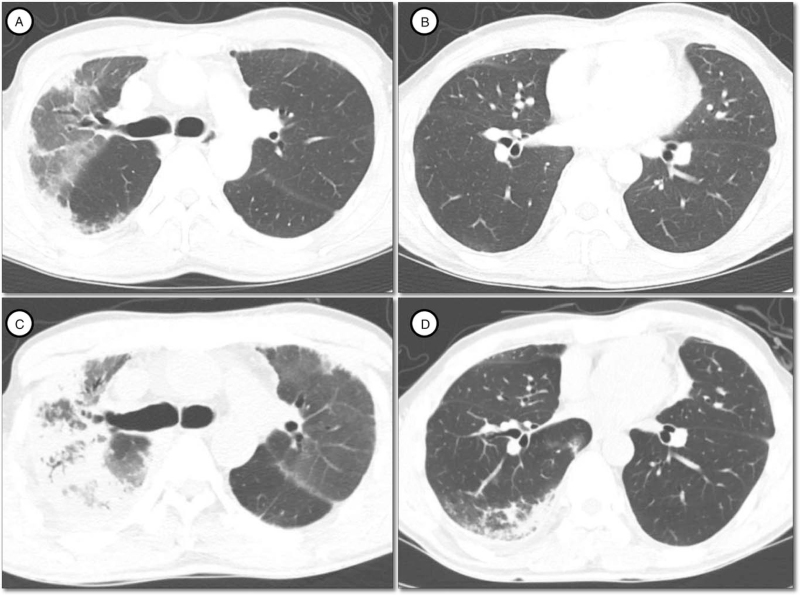
Computed tomography findings before and after the reinitiation of nilotinib. (A, B) Before the reinitiation of nilotinib, computed tomography demonstrated persistent fibrotic interstitial lung disease in the right upper lobe. (C, D) Six months after the reinitiation of nilotinib, computed tomography demonstrated aggravated subpleural consolidations with traction bronchiectasis in the right upper lobe and newly developed subpleural consolidation in the right lower lobe.

The patient was diagnosed with nilotinib-induced ILD and was treated with a prednisolone (50 mg daily). Corticosteroid treatment was continued and tapered out for 2 months. Cough was improved after the course of corticosteroid treatment. However, chest radiograph showed no significant improvement. Nilotinib (300 mg twice daily) was resumed in April 2020. Six months after the reinitiation of nilotinib, cough and progressive dyspnea were developed. Chest CT scan revealed aggravated subpleural consolidations with traction bronchiectasis (Fig. 3). After discontinuation of drug and course of corticosteroid treatment, his symptoms were improved. He is currently treated with ponatinib and has no respiratory deteriorations.

## 3 Discussion

The diagnosis of drug-induced ILD (DILD) is based on a reliable clinical judgment. Criteria for diagnosis include;

1. Use of the suspected drug,
2. Consistency with clinical and radiologic findings,
3. Exclusion of other conditions (e.g., infection, disease progression),
4. Clinical improvement after the withdrawal of the suspected drug, and
5. Clinical deterioration after the re-exposure to the drug.^[15]^

In this case, other causative drugs other than nilotinib were not identified through the medical history. The patient had no evidence of autoimmune rheumatic disease based on the laboratory findings, symptoms and clinical signs. Clinical symptoms were improved after nilotinib discontinuation and steroid treatment. Moreover, clinical and radiologic deteriorations were observed after the drug reinitiation.

In the present case, the lesion was noted in the unilateral lung. However, DILD generally presents with a bilateral lung distribution.^[16,17]^ Hence, other etiologies like malignancy and infection should be considered in a unilateral lung involvement. BAL fluid analysis showed no evidence of pulmonary infection. Surgical lung biopsy specimen was consistent with NSIP. Since drug-induced lung injury can present with various histopathologic patterns (e.g., NSIP, organizing pneumonia, diffuse alveolar damage, hypersensitivity pneumonitis and pulmonary hemorrhage),^[18]^ the diagnosis of nilotinib-induced ILD was appropriate. To the best of our knowledge, this is the first case report of irreversible nilotinib-induced ILD.

Previously, Go et al first reported a case of a 67-year-old woman patient with CML who developed ILD after 34 months of nilotinib treatment.^[19]^ CT scan revealed organizing pneumonia pattern that had predominantly bilateral subpleural consolidations. Trans-bronchial lung biopsy revealed bronchiolitis obliterans organizing pneumonia. Contrary to the current case, lung lesions were nearly recovered after the corticosteroid treatment and the patient was successfully reinitiated with nilotinib treatment without recurrence of ILD. These differences can be explained by the radiologic and pathologic patterns. While certain DILDs (e.g., organizing pneumonia, eosinophilic pneumonia and hypersensitivity pneumonitis) show good responsiveness to corticosteroid, DILD with fibrosis (e.g., idiopathic pulmonary fibrosis or idiopathic NSIP) have a poor treatment outcome.^[16]^ Moreover, it is possible that prolonged drug exposure may cause pulmonary fibrosis. In a case report of imatinib-induced irreversible ILD,^[5]^ the duration of drug exposure was longer than that reported in the previous case series.^[6]^

DILDs are uncommon, but can be fatal. Anti-cancer agents are the most common causes of DILD.^[17]^ Delayed diagnosis of DILD may result in progressive lung injury due to continuous drug exposure and delayed cancer treatment, and may contribute to poor life quality, increased medical costs, and high mortality. Hence, DILD must be considered when lung parenchymal abnormalities present in patients undergoing cancer treatment. Clinicians should be aware that nilotinib can cause irreversible fibrotic ILD, albeit rarely. Hence, there should be meticulous monitoring of the associated symptoms and radiologic findings. In addition, thorough exclusion of other differential diagnoses is mandatory.

The patient has provided informed consent for publication of the case. The study was approved by the Institutional Review Board of Chungbuk National University Hospital (No: 2021-09-032).

## Author contributions

**Conceptualization:** Jun Yeun Cho, Yoon Mi Shin.

**Data curation:** Ok-Jun Lee, Jihyun Kwon, Dohun Kim.

**Formal analysis:** Jun Yeun Cho, Yoon Mi Shin.

**Investigation:** Jun Yeun Cho, Ok-Jun Lee, Jihyun Kwon, Dohun Kim, Yoon Mi Shin.

**Writing – original draft:** Jun Yeun Cho.

**Writing – review & editing:** Ok-Jun Lee, Jihyun Kwon, Dohun Kim, Yoon Mi Shin.
